# Probiotics as a therapeutic strategy in Major Depressive Disorder

**DOI:** 10.1192/j.eurpsy.2022.1420

**Published:** 2022-09-01

**Authors:** L. Vargas

**Affiliations:** Hospital del Mar, Psychiatry, Barcelona, Spain

**Keywords:** major depressive disorder, probiotics, Treatment, gut microbioma

## Abstract

**Introduction:**

Major depressive disorder is a prevalent disease, in which one third of sufferers do not respond to antidepressants. Disturbance in the equilibrium of the gut microbiota has been involved in the pathophysiology of depression. Probiotics have the potential to be well-tolerated and cost-efficient treatment options. However, there is not enough evidence of the impact of probiotics in patients suffering MDD.

**Objectives:**

The main aim of this revision is to assess those clinical trials that evaluate the effects of probiotic treatment in patients with MDD.

**Methods:**

A research on the database PubMed has been done with the terms “probiotics” AND “MDD” and then a systematic review has been performed between those articles meeting the inclusion criteria.

**Results:**

Most of the articles show an improvement of the depressive symptoms in outpatients with mild to moderate TDM after 8 week treatment with probiotics added to the treatment as usual. Those articles assessing inpatients with severe MDD after four weeks of treatment with probiotics added to their usual treatment didn’t find statistical differences between treatment with probiotics from placebo.

**Conclusions:**

Probiotics may be useful in mild to moderate symptoms of MDD after 8 weeks treatment added to usual treatment. Nevertheless, further investigation in larger samples during more time. Moreover, a new awareness is raised about gut- brain axis pathophysiology, that would lead the path to new investigations about this relation so as the difference in depressed patients microbiome, tryptophan metabolism and the pro- inflammatory compounds that reach the blood-brain barrier because of the “leaky-gut”.

**Disclosure:**

No significant relationships.

